# Dietary phytochemicals modulate skin gene expression profiles and result in reduced lice counts after experimental infection in Atlantic salmon

**DOI:** 10.1186/s13071-016-1537-y

**Published:** 2016-05-10

**Authors:** Helle Jodaa Holm, Simon Wadsworth, Anne-Kari Bjelland, Aleksei Krasnov, Øystein Evensen, Stanko Skugor

**Affiliations:** Faculty of Veterinary Medicine and Biosciences, Sea Lice Research Centre, Norwegian University of Life Sciences, PO Box 8146 Dep, 0033 Oslo, Norway; Sea Lice Research Centre, EWOS Innovation, Dirdal, Norway; Nofima AS, Osloveien 1, Ås, Norway

**Keywords:** Atlantic salmon, *Salmo salar*, Sea lice, Anti-attachment feed, Glucosinolates

## Abstract

**Background:**

The use of phytochemicals is a promising solution in biological control against salmon louse (*Lepeophtheirus salmonis*). Glucosinolates belong to a diverse group of compounds used as protection against herbivores by plants in the family Brassicaceae, while in vertebrates, ingested glucosinolates exert health-promoting effects due to their antioxidant and detoxifying properties as well as effects on cell proliferation and growth. The aim of this study was to investigate if Atlantic salmon fed two different doses of glucosinolate-enriched feeds would be protected against lice infection. The effects of feeding high dose of glucosinolates before the infection, and of high and low doses five weeks into the infection were studied.

**Methods:**

Skin was screened by 15 k oligonucleotide microarray and qPCR.

**Results:**

A 25 % reduction (*P* < 0.05) in lice counts was obtained in the low dose group and a 17 % reduction in the high dose group compared to fish fed control feed. Microarray analysis revealed induction of over 50 interferon (IFN)-related genes prior to lice infection. Genes upregulated five weeks into the infection in glucosinolate-enriched dietary groups included Type 1 pro-inflammatory factors, antimicrobial and acute phase proteins, extracellular matrix remodeling proteases and iron homeostasis regulators. In contrast, genes involved in muscle contraction, lipid and glucose metabolism were found more highly expressed in the skin of infected control fish.

**Conclusions:**

Atlantic salmon fed glucosinolates had a significantly lower number of sea lice at the end of the experimental challenge. Feeding glucosinolates coincided with increased expression of IFN-related genes, and higher expression profiles of Type 1 immune genes late into the infection. In addition, regulation of genes involved in the metabolism of iron, lipid and sugar suggested an interplay between metabolism of nutrients and mechanisms of resistance.

**Electronic supplementary material:**

The online version of this article (doi:10.1186/s13071-016-1537-y) contains supplementary material, which is available to authorized users.

## Background

Sea lice infections constitute a major and global problem for salmonid aquaculture. Infection control relies primarily on chemical treatments whose repertoire is limited because of resistance to existing anti-parasitic compounds [1]. In addition, stress to the fish caused by frequent delousing events is of particular concern [2]. In order to apply the optimal treatment, resistance monitoring in lice populations, implemented through a nationwide surveillance program in Norway, could be helpful [1–4]. The amount of chemotherapeutants used against lice in Norway has surged over recent years; in 2008, 218 kg (kg of active substance, excluding hydrogen peroxide) were used, compared to 6,810 kg in 2012, 8,403 kg in 2013 and 12,812 kg in 2014 [5]. Despite differences in the dosage of used chemotherapeutants and increase in the general production of Atlantic salmon, the rise in the number of treated salmon is likely the result of resistance development in sea lice [3, 6]. Finding alternative strategies for managing lice infections is therefore becoming increasingly more important.

Salmon lice (*Lepeophtheirus salmonis*) immunomodulate their hosts by secreting a complex cocktail of bioactive compounds [7–9]. Release of these secretory/excretory products depends on the host species, being highest in response to Atlantic salmon [10]. This is in line with comparative studies showing that Atlantic salmon is among the most susceptible salmonid species [11–16]. Activation of the Type 1 immunity, similar to mammalian Type 1 (Th1 and Th17 responses), could play a role in the resistance of salmonids to lice infections [12, 13, 17, 18], especially during early stages of infection [12]. Pro-inflammatory Type 1 responses (and to a lesser extent Type 2 responses) in skin were negatively correlated to the number of *L. salmonis* chalimus stages in Atlantic salmon [17]. Skewing of immunity towards the Type 2 immunophenotype with a strong immunosuppressive component in Atlantic salmon (termed Th2-modified) likely contributes to susceptibility of Atlantic salmon [18], while Type 2 responses seem to have a more beneficial role in coho salmon during later stages of infection with *L. salmonis* [12].

There are several encouraging examples of the use of orally delivered microbial immunostimulants that promote protective immune responses [19–22]. The use of dietary plant-derived bioactives is also considered a promising approach. Plants in the family Brassicaceae contain secondary metabolites called glucosinolates (GLs) that protect against herbivory [23], bacterial and fungal disease agents [24–26]. When plant cells are destroyed by chewing or other mechanical processing, the enzyme myrosinase comes into contact with GLs and hydrolyses them into isothiocyanates (ITCs). These compounds act as insect deterrents but might also be toxic to invertebrates upon ingestion [25, 27]. Their strong pungent flavor [28] may also mask the host smell and obscure the host recognition and/or attachment process by sea lice. A range of olfactory receptors have been identified in both *L. salmonis* and *Caligus rogercresseyi* [29–31].

Studies in mammals have revealed that GLs-derived ITCs exert chemopreventive effects mainly attributed to induction of antioxidant and detoxification pathways (reviewed in [32, 33]). A majority of in vivo and in vitro studies report anti-inflammatory effects of ITCs in a range of pathological conditions, organs and cell lines, including tumor cells [32, 34]. However, pro-inflammatory type 1 responses have also been seen in murine skin after exposure to ITCs [35], suggesting an organ dependent regulation of immune responses by ITCs.

To date, GLs and their breakdown products have not been investigated as feed additives against aquatic parasites of Atlantic salmon. In this study, we hypothesised that dietary GLs would modulate skin immune and physiological responses, prior to and during lice infection, thus interfering with the attachment and establishment of *L. salmonis*.

## Methods

### Ethics statement

The experimental facilities used in this study at Ewos Innovation, Dirdal, Norway, number 131 was approved by the Norwegian Animal Research Authority 02.02.2012 until 25.01.16. The experiments/procedures have been conducted in accordance with the laws and regulations controlling experiments/procedures in live animals in Norway, e.g. the Animal Welfare Act of 20th December 1974, No 73, chapter VI sections 20–22 and the Regulation on Animal Experimentation of 15th January 1996.

### Fish trials, production of feeds and copepodids

All trials were performed at Ewos Innovation’s Test Facility in Dirdal, Norway from October to December 2012. Fish tanks used in all trials were 500 l circular flow-through tanks with an average temperature and salinity of 8.7 °C and 27.4 ppt, respectively.

The feeds used in this study were produced at the Ewos Innovation plant in Dirdal, Norway. The fish were fed with the control feed (C) OPAL (EWOS Opal, EWOS, Norway) and two experimental (anti-attachment) feeds that contained GLs. The low dose feed (LD) had 3.61 % and the high dose feed (HD) had 13.0 % of the GLs-containing ingredient originating from a plant of the family Brassicaceae, with the approximate GLs content of 7.3 μmol/g and 26.4 μmol/g in LD and HD, respectively (see Additional file 1: Table S2 for details of the dietary composition). All diets had a pellet size of 5.5 mm.

**Fig. 1:**
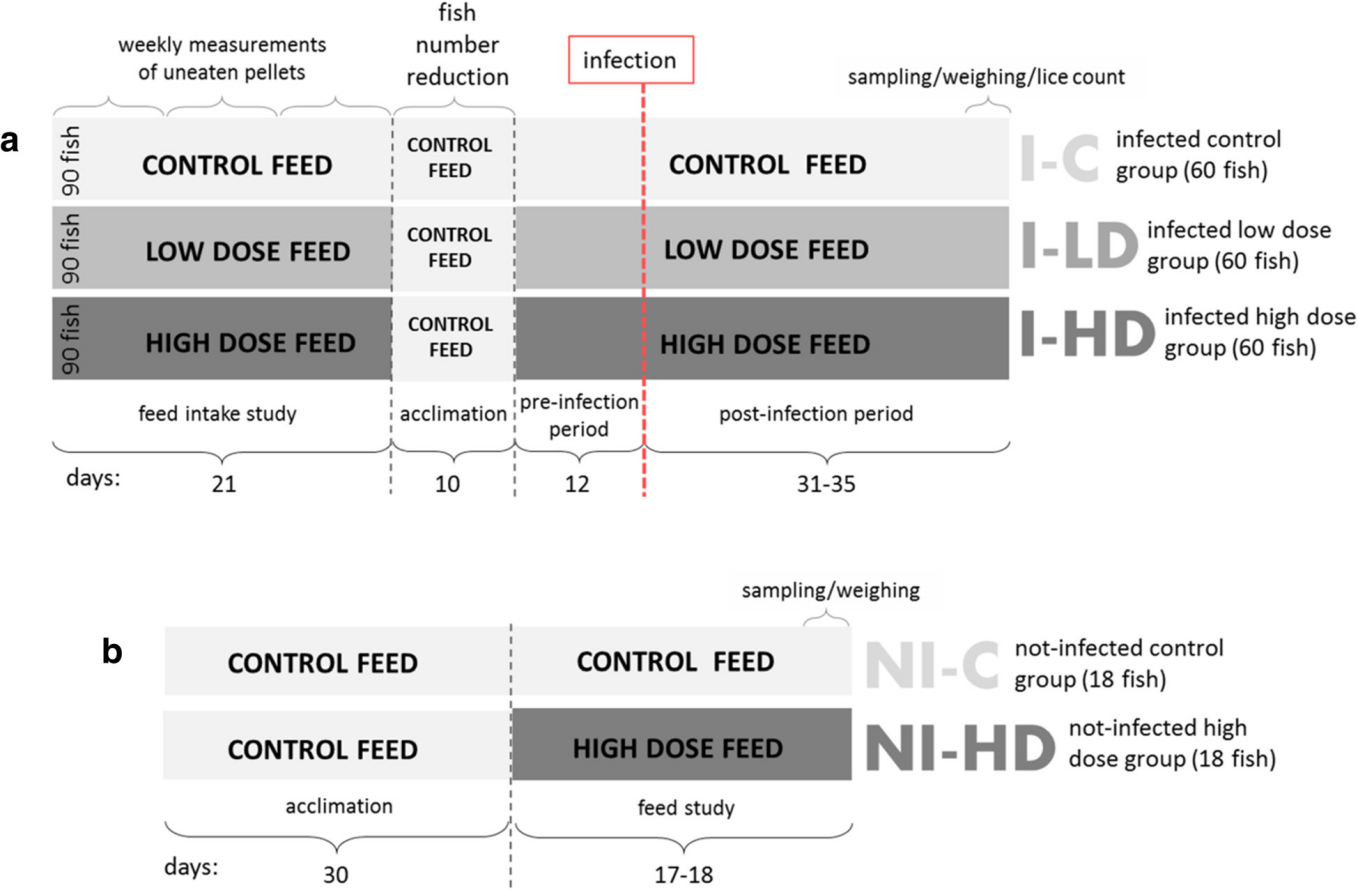
Experimental trial setup. **a** Feed intake and lice challenge study. Atlantic salmon were fed control feed, low dose and high dose level of GLs for 21 days. The fish were then fed control feed for 10 days (acclimation), before continuing on the control, low dose and high dose feeds for 12 days (pre-infection period). The skin samples, weighing of the fish and lice counting from the three dietary groups challenged with *L. salmonis* (50 copepodites per fish) [infected fish fed control feed (I-C), infected fish fed low dose feed (I-LD) and infected fish fed high dose feed (I-HD)] were done after 31–35 days of infection (post-infection period). **b** Feed study. To study responses of the feed *per se* (without infection), Atlantic salmon were fed control feed and high dose feed. All fish were acclimatised with the same control feed for 30 days before the feed study commenced. Sampling of skin tissue and weighing of the fish from two groups [not-infected control group (NI-C) and not-infected high dose group (NI-HD)] were performed after 17–18 days of feeding experimental feeds

To examine the feed intake for the three diets, 30 fish in three tank replicates (90 per diet) were fed to satiation for three weeks (Fig. 1a). The amount of uneaten pellets was measured weekly. After the feed intake study, all tanks were fed OPAL and the numbers of fish in each tank was reduced from 30 to 20 (acclimation period). After 10 days of control feeding, six of the tanks were fed the anti-attachment LD and HD feeds and three tanks continued on C for 12 days (pre-infection period), and throughout the 31–35 days of *L. salmonis* infection (post-infection period). The treatment groups tested in this part of the study were named: infected C (I-C) infected LD (I-LD) and infected HD (I-HD) in three tank replicates. After feeding the experimental diets for 12 days (pre-infection period), fish were infected with 50 copepodids per fish by turning off the water flow and lowering water level to 15 cm height before copepodids were evenly distributed to the nine fish tanks. Oxygen was added using a fine ceramic diffusor, with individual air valves controlling the oxygen flow to each tank. After 1 h of exposure, water flow was resumed. Lice counting and sampling were done when majority of lice reached preadult stages. During a sampling period of four days, number, stage and gender of lice on each fish were recorded. In addition, skin samples from 3 fish from each tank (9 fish from each group), approximately 5 × 5 mm in size, were excised from the site immediately caudally of the dorsal fin, and put in RNA*later* (Ambion®, Austin, TX, USA) at 4 °C for 24 h and then stored at -80 °C until further processing. Fish weights and lengths, and the presence of feces were also registered. Lice counts were analysed by one-way ANOVA with Tukey’s *post*-*hoc* test using the GraphPad Prism 6.0 software. Fish performance and distribution of life stages were analysed by using Microsoft Excel 2010. Fulton's condition factor was calculated by the formula: (100 BWFL^-3^) [36].

Six tanks of fish were used in a parallel feed study (Fig. 1b) to assess the effect of GLs feeding *per se* (without infection). After one month of feeding control feed (acclimation), three tanks of fish were fed high dose (HD) diet. The other three tanks continued on control feed. These groups of fish were named not-infected high dose (NI-HD) and not-infected control (NI-C), respectively. Sampling of skin tissue was performed after 17–18 days of feeding using the same protocol as for the infected fish; skin tissue of 9 fish from each group were sampled, and weights and length of 18 fish from each group were registered.

Salmon lice (*L. salmonis*) used in this trial were collected from Oltesvik (Norway) in March 2012. To provide a predictable supply of lice for future trials, this lice population was propagated and maintained on Atlantic salmon hosts kept in the *L. salmonis* cultivation system in the Sea Lice lab at the Dirdal facility, which provided stable supply of robust wild-type lice. Lice and host fish were held in 850 l circular flow-through tanks and egg strings from egg-bearing females were collected from anaesthetised salmon. The anesthetic used was Finquel (100 mg/l, Scan Aqua, Årnes, Norway). During an incubation period of 14 days (9 °C), the egg strings were allowed to hatch and reach the infective copepodid stage. The number of copepodids was counted in a zooplankton-counting chamber to calculate the density. At least four samples of 50 ml each were taken to improve the accuracy of estimation.

### RNA extraction and cDNA synthesis

Total RNA extraction was done using the RNAeasy Mini Kit (Qiagen, Hilden, Germany) after adding Trizol (GIBCO, Life Technologies, Carlsbad, CA, USA) and homogenizing 50 mg of samples with 1.4 mm zirconium oxide beads (VWR, Oslo, Norway). After this, chloroform was added, samples were centrifuged and the RNA supernatant was subsequently subjected to RNA cleanup according to Qiagen protocol. The concentration of RNA was determined by spectrophotometry using NanoDrop ND1000 (Nanodrop Technologies, Wilmington, DE, USA) and stored at -80 °C until further use. The integrity of total RNA was determined using an Agilent 2100 Bioanalyzer, and only samples with RNA integrity number (RIN) of 8 or higher were accepted. Genomic DNA contamination was excluded by performing qPCR reactions using isolated RNA as templates together with primers for elongation factor-1*α* (*EF1A*).

### Microarray hybridization and data processing

Five fish from each group (I-C, I-LD, I-HD and NI-C), and four fish from NI-HD were analysed by 15 k Atlantic salmon microarray (SIQ6); these individuals were a subset of fish analysed by qPCR. All samples were compared to pooled reference RNA that consisted of two fish from all groups, except for I-LD. The test samples labelled with Cy5 and pooled reference with Cy3 were competitively hybridised to array slides. All reagents and equipment used for microarray analyses were from Agilent Technologies; protocols were used according to the manufacturer. Labelling and amplification of RNA was performed on 100 ng total RNA using Two-colour Quick Amp Labelling kits. Gene Expression Hybridization Kit was used for the fragmentation. Hybridizations were performed in a rotation oven for 17 h at 65 °C with rotation speed of 10 rpm, followed by 1 min washing of arrays with Gene Expression Wash Buffer I at room temperature and Gene Expression Wash Buffer II at 37 °C. To achieve an overall intensity ratio close to 1 between Cy3 and Cy5 channels with minimal saturation, the slides were scanned immediately using GenePix Personal 4100A scanner (Molecular Devices, Sunnyvale, CA, USA) at 5 μm resolution and with manually adjusted laser power. For feature extraction of fluorescence intensity values and assessment of spot quality that followed spot-grid alignment, the GenePix pro software 6.0 was used. Subsequent to filtration of low quality spots flagged by the software, Lowess normalization of log_2_-expression ratios (ER) was performed. Differentially expressed genes (DEGs) were selected by comparison with the not-infected control (NI-C): log_2_-ER > 0.6 and *P* < 0.05 in at least one group were the criteria used. Fold values of log_2_-ERs (DEGs) were then calculated. Nofima’s bioinformatics system (STARS) was used for data analyses.

### qPCR protocol

To validate the microarray data and screen other genes of interest (see Additional file 1: Table S1 for the full list), 9 fish from each group (I-C, I-LD, I-HD and NI-HD), in addition to NI-C group of fish were analysed by qPCR. For each sample, 1,800 ng of RNA was used to synthesize cDNA using the cDNA Affinity Script (Agilent Technologies, Matriks AS, Oslo, Norway) following the manufacturer’s protocol. Every reaction contained 1 μl of random primers and 2 μl of oligo DT primers. Each gene was run in duplicates by adding 4 μl of 1:10 diluted cDNA from each fish, 1 ul of each primer (10 μM concentration) (see sequences in Additional file 1: Table S1) and LightCycler 480 SYBR Green I Master mix (Roche) to a final volume of 12 μl in 96-well plates. Cycling conditions in LightCycler 480 instrument (Roche, Applied Science) were 5 min denaturation step at 95 °C, 40 cycles of denaturation (10 s at 95 °C), annealing (20 s at 60 °C) and extension (15 s at 72 °C), followed by melting curve analysis with measurements of the fluorescence performed in the temperature range between 65–97 °C. The maximum-second-derivative method (Roche diagnostics) was used to find the crossing point (Cp) value. The relative expression of target genes was calculated by using the ΔΔCt method. The reference gene *EF1A* was selected as it is one of the most well-established reference gene in studies of Atlantic salmon tissues in general [37] as well as in lice infected tissues [12, 17, 21, 38–40]. In this study, the mean Cp value in each group varied less than 0.5 cycle. One-way ANOVA with subsequent Tukey’s multiple comparisons test in the GraphPad Prism Software were executed between each of the 5 groups. Specificity and efficiency were confirmed by melting curve analysis and two-fold serial dilutions of cDNA for each primer pair in triplicates, respectively. PCR efficiency for all genes ranged from 1.8–2.

## Results

### Fish performance and lice counts

No changes in appetite were observed for any of the diet groups during the study period. Only two fish from the control group died during the trial period (of non-specific causes). The fish weights [mean (g) ± SD] at the end of the lice-challenge (Fig. 1a) were as follows: I-C: 871 ± 127; I-LD: 751 ± 121; and I-HD: 726 ± 113, where the two latter groups differed significantly from the control group (I-LD *vs* I-C: *t*-test: *t*_(38)_ = 3.62, *P* = 0.0009; I-HD *vs* I-C: *t*-test: *t*_(38)_ = 4.96, *P* < 0.0001). However, the condition factor (mean ± SD) was lower in control than in fish exposed to GLs: I-C group (1.43 ± 0.13), I-LD (1.54 ± 0.16) and I-HD (1.52 ± 0.13) (I-LD *vs* I-C: *t*-test: *t*_(38)_ = 2.255, *P* = 0.030; I-HD *vs* I-C: *t*-test: *t*_(38)_ = 1.65, *P* = 0.10). In the feed study (Fig. 1b), neither weights nor condition factors differed significantly between the NI-C group and NI-HD (weight *t*-test: *t*_(34)_ = 0.49, *P* = 0.62; condition factor *t*-test: *t*_(34)_ = 1.37, *P* = 0.18). Lice counts and tissue sampling were performed at five weeks post-infection, when most of the female lice had reached preadult 2 stage. The distribution of different life stages was not affected by the diet (Table 1).

**Table 1:** Distribution of gender and life stages of lice found on Atlantic salmon in the infected control group (I-C), infected group fed low inclusion level of GLs (I-LD) and infected group fed high inclusion level of GLs (I-HD)

Dietary group/stage	Preadult 1 (males)	Preadult 1 (females)	Preadult 2 (males)	Preadult 2 (females)	Adult (males)
I-C	0.55	2.02	8.46	46.18	42.78
I-LD	0.37	2.48	7.43	46.53	43.19
I-HD	0.11	1.33	8.23	46.83	43.38

Variables are shown as percentages of the total (100 %) lice count for each group

Lice were counted on 180 fish (60 fish per dietary group), and the average number of lice ± SD was 15.4 ± 5.3 (range 4–35). The mean number of lice in each group was I-C: 18 ± 5.1; I-LD: 13.5 ± 4.8; and I-HD: 15 ± 4.6. Compared to infected control fish (I-C), there was a 25 % reduction in lice number in the I-LD group, while I-HD had 17 % less lice (Table 2). One-way ANOVA with Tukey’s *post*-*hoc* test (ANOVA: *F*_(2, 177)_ = 14.39, *P* < 0.0001) showed significant difference in lice number between I-LD and I-C (*P* < 0.0001), as well as between I-HD and I-C (*P* = 0.0036).

**Table 2:** Examples of immune genes with differential expression in skin (microarray results)

Gene	Abbreviation	Accession	NI-HD	I-C	I-LD	I-HD
Chemokines, cytokines and receptors						
C-C motif chemokine 19-1	*CCL*-*C5A*	209737465	2.87	1.17	1.53	2.03
C-C motif chemokine 19-2	*CCL*-*C5A*	117433169	2.87	1.15	1.49	1.95
C-C motif chemokine 13		EG872936	2.33	1.39	1.41	1.87
CXCL10-like chemokine		EF619047	3.73	-1.40	-1.04	1.35
Leukocyte cell-derived chemotaxin 2-1	*BX005069.2*	117545301	1.04	5.76	2.47	7.35
Small inducible cytokine A13	*CCL13*	GE835061	2.67	1.02	1.48	2.65
C-C chemokine receptor type 3	*CCR12.3*	223648789	2.11	1.20	1.47	1.97
Interleukin-20 receptor alpha chain	*CRFB2*	117446659	4.94	1.07	1.17	1.38
Effectors						
TNFa induced metalloreductase STEAP4		223649457	1.58	-1.19	2.12	1.53
Neutrophil cytosolic factor 1	*NCF1*	223647567	1.31	1.32	2.17	2.03
RNase 1	*RNASE1*	DN047839	-1.10	8.63	2.74	11.34
Granzyme A	*GZMA*	209733889	2.95	-1.01	1.01	1.74
Complement factor H1 protein	*CFH1*	DY713380	1.01	6.02	1.95	7.39
Antimicrobial peptide NK-lysin		EG840346	2.93	1.61	2.41	1.68
Natterin-like protein	*NATTL*	223584499	-1.01	1.08	2.29	2.50
C1q-like specific protein	*CBLN8*	117537786	9.46	21.86	31.38	106.26
Cathelicidin antimicrobial peptide 2	*CATH*-*2*	AY360357	1.87	2.20	3.62	3.46
Collagenase 3	*MMP13A*	AJ424540	3.21	3.66	7.63	5.22
MMP 13 or Collagenase 3	*MMP13A*	209156091	2.97	2.67	7.34	4.57
Lectins and coreceptors						
Mannose-specific lectin		209733483	2.68	3.31	6.34	9.57
P-selectin	*SELE*	BT058751	2.53	1.28	1.45	1.81
C type lectin receptor A		AY572832	2.17	2.55	3.18	2.74
Leukolectin protein		60377755	1.26	2.51	1.35	-1.11
CD83	*CD83*	DQ339141	2.13	1.01	-1.28	1.25
CD97 antigen	*CABZ01066772*	EG935955	1.81	1.22	1.55	1.92
CD209 antigen-like protein E		S35562993	2.17	-1.07	-1.03	1.00

Data are mean fold calculated from log_2_ (ER) values and compared to NI-C. Values with significant difference compared to the NI-C group are underlined

### Gene expression responses to GLs, lice and their combinations: microarray analyses

**Fig. 2:**
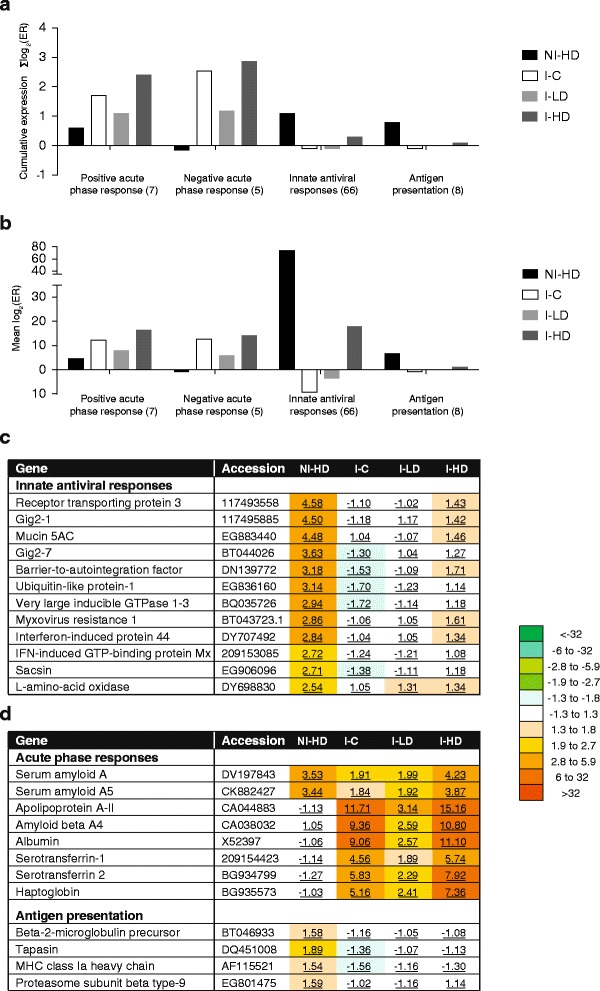
Immune genes with correlated expression profiles (microarray results). **a** Cumulative expression changes assessed as Σlog_2_ (ER). **b** Mean log_2_ (ER); numbers of features are in parentheses. **c** Tabulated examples of most regulated genes involved in innate antiviral responses. Data are mean fold calculated from log_2_ (ER) values and compared to NI-C (significantly different values are underlined. **d** Tabulated examples of the most regulated genes involved in positive and negative acute phase response and antigen presentation. Data are mean fold calculated from log_2_ (ER) values and compared to NI-C (significantly different values are underlined)

**Fig. 3:**
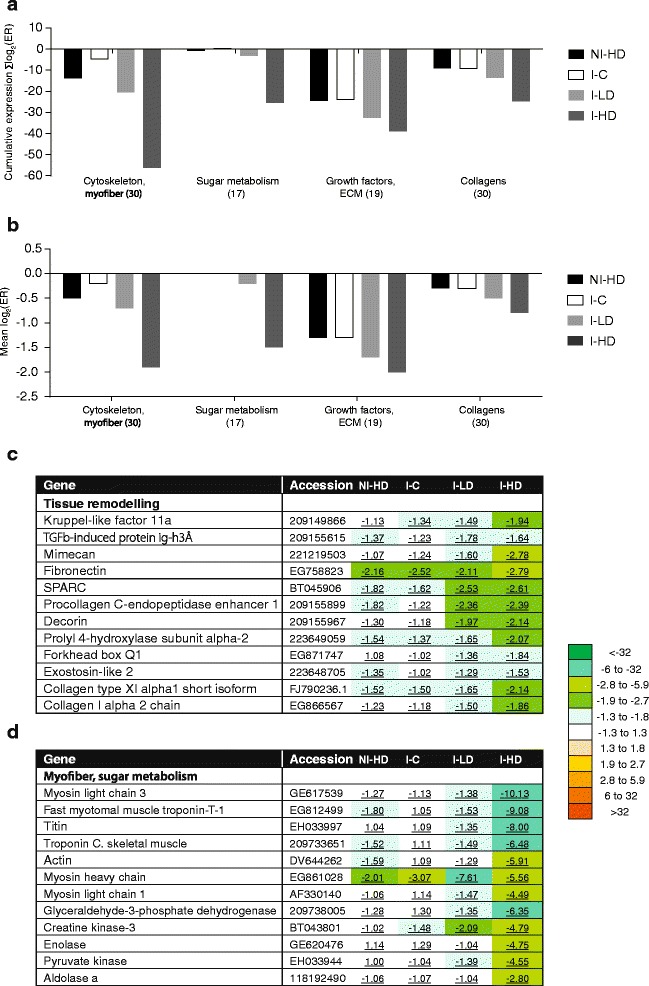
Genes encoding cytoskeletal and myofiber (contractile) proteins, enzymes involved in sugar metabolism, growth factors and collagens with correlated expression profiles (microarray results). **a** cumulative expression changes assessed as Σlog_2_ (ER); **b** mean log_2_ (ER); numbers of features are in parentheses. **c** Tabulated are examples of the most regulated genes involved in myofiber and sugar metabolism. Data are mean fold calculated from log_2_ (ER) values and compared to NI-C (significantly different values are underlined). **d** Tabulated examples of most regulated genes involved in tissue remodeling. Data are mean fold calculated from log_2_ (ER) values and compared to NI-C (significantly different values are underlined)

In GLs-containing feed groups, lice infection resulted in the increased expression of multiple genes and a large part of the upregulated genes was categorised as immune genes. A similar trend of expression among genes that were grouped according to their biological function was found within, but not between the treatments (Figs. 2 and 3). The largest group (66 features, Fig. 2a-c) consisted of genes associated with innate antiviral immunity [41]. In this study, innate antiviral genes were strongly upregulated in the not-infected GLs-group (NI-HD) compared to NI-C, lice infection resulted in slight downregulation in I-C, while I-LD and I-HD showed intermediate values (Fig. 2c). One example is myxovirus resistance 1 that was upregulated 2.86-fold in NI-HD, downregulated following lice infection in I-C, while being significantly increased (1.61-fold) in I-HD (Fig. 2c). Receptor transporting protein 3 was on the top of the list with 4.6-fold upregulation in NI-HD compared to NI-C (Fig. 2c). To note was also the concerted induction of several GTPases and GTP binding proteins, which are known as important components of the cellular antiviral response. Slight GLs-mediated induction of genes involved in antigen presentation was suppressed after lice infection, with no recovery five weeks into the infection when most lice reached the preadult stage (Fig. 2d).

Activation of the acute phase response genes in skin included increased expression in NI-HD and I-HD of serum amyloid A, A5 and amyloid beta A4 compared to NI-C (Fig. 2d). On the other side, lowest expression of genes encoding proteins with diverse transport and scavenger functions (many of which are classified as negative acute phase plasma proteins) was found in I-LD (Fig. 2d). Most other immune genes were also induced, either by GLs, lice or both, when compared to NI-C (Table 2).

Majority of genes affected by GLs in infected groups showed dose-dependent responses: changes in I-HD were either equal to or greater than I-LD. However, several matrix metalloproteinase (*MMP*) genes, critical for extracellular remodeling during wound healing and inflammation [42] reached maximum expression levels in I-LD. Increased *MMP13* expression has been linked to lice resistance in our previous study [17]. In this study, C1q-like specific protein showed the overall greatest expression change (31.38-fold) in the best-protected I-LD group compared to NI-C. Natterin-like protein (*NATTL*) is a homologue of *NATTL* gene in *T. nattereri* that codes for a protein found in the venomous secretions of this fish species [43] and neutrophil cytosolic factor 1 were stimulated only by combinations of GLs and lice but at much lower levels. The upregulated immune genes included a number of inflammatory mediators. Two C-C motif chemokines 19 (*CCL19*), which responded to GLs might have a role in T cell proliferation and maturation of DCs that promote Th1 rather than Th2 responses [44]. Similar profile was seen for several other chemokines and receptors (Table 2). A Th2 marker C-C chemokine receptor type 3 [45] was more highly expressed in GLs groups, a similar trend was shown by granzyme A, an effector molecule of T cells. Furthermore, the small inducible cytokine A13 (*CCL13*) (2.65-fold induced) is a chemoattractant for a diverse group of immune cells [46]. High (5-fold) induction of IL-20 receptor alpha chain precursor (4.95-fold) in NI-HD, followed by 1.38 fold in I-HD was noteworthy as this receptor transduces highly pro-inflammatory signals in mammalian skin [47]. The leukocyte cell-derived chemotaxin 2 was upregulated by lice (I-C) and even more so in I-HD, and is associated with responses to lice [18] and lice resistance [17] in our previous studies.

Genes for diverse innate effectors were also activated. Golgi-residing metalloreductase STEAP family member 4, associated with iron metabolism and inflammation [48, 49] responded only to GLs, being most highly induced in I-LD, suggesting links between iron regulation, inflammation and resistance to lice. The antimicrobial peptides NK-lysin and cathelicidin antimicrobial peptide 2 that have been correlated to lice resistance in our previous studies [17, 18, 39] were stimulated by both diet and lice infection, and their combination, while RNase 1, a cell-cidal effector and complement regulatory factor H1 were upregulated only by lice infection (I-C). Several lectins and lectin co-receptors present on leucocytes were induced by GLs (p-selectin, *CD83*, *CD209* and *CD97*) or by a combination of lice and GLs (mannose-specific lectin and C type lectin receptor A).

Apoptotic and stress responses to GLs and lice in skin of Atlantic salmon were relatively weak (Table 3). Caspase-3 showed slight induction, while synergistic downregulation was observed for pro-apoptotic ` switch protein 2 [50]: 4-fold in I-LD compared to NI-C and 4.3-fold in I-HD. Upregulation by both lice and GLs was seen in two genes encoding heat-shock proteins and glutathione peroxidase, a scavenger of free radicals. Hydrogen peroxide producing enzyme *L*-amino-acid oxidase was induced only by GLs.

**Table 3:** Examples of genes with differential expression in skin involved in apoptosis, stress responses, cytoskeleton and steroid and lipid metabolism (microarray data)

Gene	Abbreviation	Accession	NI-HD	I-C	I-LD	I-HD
Apoptosis and stress						
G0/G1 switch protein 2	*G0S2*	117545986	-1.41	1.24	-3.96	-4.28
Caspase 3A	*CASP3B*	S24639607	1.33	1.39	1.54	1.73
60 kDa heat shock protein, mitochondrial	*HSPD1*	223649223	1.39	1.50	1.86	1.97
Heat shock protein 4	*HSPA4B*	DY713457	1.38	1.19	1.69	1.97
Glutathione peroxidase type 2	*GPX1A*	CA345885	1.50	1.50	2.02	1.79
L-amino-acid oxidase		DY698830	2.54	1.05	1.31	1.34
Cytoskeleton						
Keratin 12		EG798776	1.28	1.80	1.66	2.38
Keratin type I cytoskeletal 17		DY692568	1.29	1.24	1.36	2.60
Type I keratin S8		CX357672	1.12	1.34	1.88	1.77
Type II keratin E3	*KRT4*	EG778421	1.09	1.27	1.48	2.57
Steroid and lipid metabolism						
Lipoprotein lipase	*LPL*	EG838215	-1.25	1.28	-1.97	-2.46
Fatty acid-binding protein, adipocyte	*FABP11A*	209735153	-1.19	1.29	-2.96	-3.01
Sex hormone-binding globulin beta	*GAS6*	DY699233	-2.00	1.23	-3.68	-5.64

Data are mean fold calculated from log_2_ (ER) values and compared to NI-C. Values with significant difference compared to NI-C group are underlined

Differential expression was seen in multiple genes encoding intracellular fibrous structural proteins. Joint treatments (diet and lice infection) induced several genes involved in keratinization (Table 3). Keratin type I cytoskeletal 17 and type II keratin E3 are parts of the epithelial cytoskeleton, which provides mechanical resilience of epithelial cells and in addition can be involved in intracellular signaling [51]. Many more genes were downregulated and several functional groups showed highly coordinated expression changes (Fig. 3a, b). Two clusters of co-expressed genes included myofiber proteins and enzymes of sugar metabolism (30 and 17 features, respectively); higher concentration of GLs produced stronger downregulation in both groups. Myofiber genes included mainly components of the myocontractile apparatus: myosin light and heavy chains, actin, troponins and titin (Fig. 3a, b, d). Similar though weaker changes were observed in lipid and steroid metabolism (Table 3). A number of genes with roles in tissue differentiation, formation of extracellular matrix (ECM) and wound healing, including multiple collagens, transcription factors forkhead box Q1 and kruppel-like factor 11a, receptor exostosin-like 2 [52, 53] and transforming growth factor-beta-induced protein ig-h3 [54] were downregulated by both GLs and lice infection (Fig. 3c). The effect was slightly enhanced by the combination of lice and high dose of GLs.

### qPCR results

**Fig. 4:**
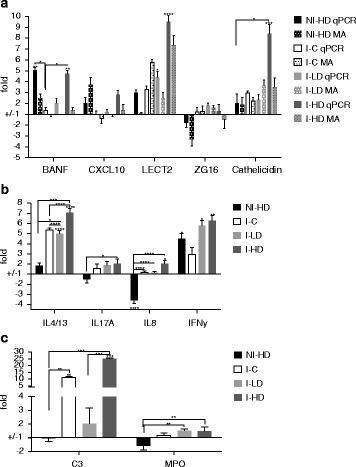
Relative gene expression analysed by qPCR and microarray (MA) in skin behind dorsal fin from four groups of *L. salmonis*-infected Atlantic salmon fed different diets; not-infected high dose (NI-HD), infected control (I-C), infected low dose (I-LD) and infected high dose (I-HD). Relative gene expression is presented as ± fold difference. Bars represent mean fold ± SEM compared to not-infected control fish, NI-C. Number of fish in each qPCR group is 9 and 5 in MA analysis. One-way ANOVA followed by post-hoc Tukey’s multiple comparisons tests was performed between groups and control. Asterisks above bars denote significant differences between groups and control: ****P < 0.0001; ***P < 0.001; **P < 0.01; *P < 0.05. Joined brackets show significant differences between experimental groups. The average correlation coefficient between MA and qPCR data was 0.8. **a** Relative gene expression of barrier-to-autointegration factor 1 (*BANF*), *CXCL10*, leukocyte cell derived chemotaxin 2 (*LECT2*), Zymogen granule membrane protein 16 (*ZG16*) and Cathelicidin-derived antimicrobial peptide 2 (Cathelicidin) . **b** Relative gene expression analysed by qPCR of interleukin 4/13 (*IL4*/*13*), interleukin 17A (*IL17A*), interleukin 8 (*IL8*), and interferon gamma (*IFN*γ). **c** Relative gene expression analysed by qPCR of complement C3 (*C3*) and myeloperoxidase (*MPO*)

Real time qPCR results are shown in Fig. 4. Expression of genes of interest are shown in Fig. 4b, c and the differential expression of *BANF* (ANOVA: *F*_(4,40)_ = 7.350, *P* = 0.0002), *CXCL10* (ANOVA: *F*_(4,40)_ = 3.147, *P* = 0.0243), *LECT2* (ANOVA: *F*_(4,40)_ = 7.171, *P* = 0.0002), *ZG16* (ANOVA: *F*_(4,40)_ = 2,214, *P* = 0.0848) and cathelicidin (ANOVA: *F*_(4,40)_ = 5.421, *P* = 0.0014) measured by the array, were validated by qPCR (Fig. 4a). Both diet and lice infection modulated skin transcriptional responses related to immunity. For most pro-inflammatory genes expression was lowest in I-C (Fig. 4b). qPCR analysis confirmed that fish groups exposed to GLs-diets had a significantly higher increase in interferons namely *IFNy*, compared to the NI-C group (ANOVA: *F*_(4,40)_ = 4.377, *P* = 0.0050; NI-HD *vs* NI-C: *P* = 0.04; I-LD *vs* NI-C: *P* = 0.01; I-HD *vs* NI-C: *P* = 0.0070). The almost double increase in the I-HD group of complement component *C3* [55] (Fig. 4c) (ANOVA: *F*_(4,40)_ = 9.761, *P* < 0.0001; I-C *vs* I-HD: *P* = 0.76) and neutrophil attractant *IL8* [56] (Fig. 4b) (ANOVA: *F*_(4,40)_ = 19.24, *P* < 0.0001; I-C *vs* I-HD: *P* = 0.145), compared to I-C group was also observed. Expression of the neutrophil marker myeloperoxidase (*MPO*) [57] (Fig. 4c) (ANOVA: *F*_(4,40)_ = 5.3, *P* = 0.0016) and neutrophil chemoattractant *IL17A* [58, 59] (Fig. 4b) (ANOVA: *F*_(4,40)_ = 3.088, *P* = 0.026) was remarkable; they were both suppressed in NI-HD but most highly induced upon infection in fish exposed to GLs-enriched feeds. Interestingly, *IL4*/*13*, a putative Th2 cytokine in fish [60] (Fig. 4b) was found to be most highly induced in I-HD (ANOVA: *F*_(4,40)_ = 19.66, *P* < 0.0001; I-HD *vs* NI-C: *P* < 0.0001), likely suggesting the need to counteract Type 1 immunity and fine tune highly pro-inflammatory immune responses.

## Discussion

The use of anti-attachment feeds promises to be a safe, easy to administer and cost-effective approach against sea lice. The achieved reduction in parasite numbers amounted to 25 % and could thus only be complementary to other control measures within the integrated pest management. Further work is needed to determine the optimal dosage and other possible effects that the bioactive compounds contained in anti-attachment feeds might exert on fish.

The goals of this study were to screen the transcriptomic response in skin after feeding Atlantic salmon diets enriched with GLs as well as to examine the impact of two inclusion doses of GLs (LD and HD) on the outcome of lice infection. One of the key findings in this study was the massive upregulation of a large group of genes involved in or associated with innate antiviral responses [41] in NI-HD (Fig. 2a-c) (the fish in this study showed no apparent signs of any viral disease). This is of note as the suppression of antiviral pathways by lice has been repeatedly reported [12, 16–19, 38, 40]. Innate anti-viral genes are co-regulated with interferons that play a key part in regulation of antiviral and antibacterial responses [61]. Stimulation of mice with ITCs resulted in increased expression level of canonical Th1 markers *IFN*γ and *T*-*bet* in the ear tissue [35]. Possible association between antiviral gene expression and reduced level of lice infection was shown in two of our previous studies. One addressed protection by sex steroid hormones, which conferred a 50 % reduction in lice counts [39] while in the other one, selective breeding based on 150 tested families resulted in a difference of around 36 % in lice counts between the top five extremely susceptible and resistant families included in the study [17]. The observations from the current study fit a previously suggested hypothesis, which states that responses similar to mammalian Type 1 pro-inflammatory responses (Th1/Th17) play a positive role in protection against *L. salmonis* in Atlantic salmon [11, 12, 18, 40, 62]. Importantly, the induction of antiviral genes observed in NI-HD also remained higher in *L. salmonis*-challenged fish fed GLs (I-HD, I-LD) five weeks post-infection.

A recent comparative transcriptomic study of pink, chum and Atlantic salmon found downregulation of antiviral immune genes in both the resistant (pink) and susceptible species (chum), thus drawing attention to other protective mechanisms in Pacific salmonids, centered around iron metabolism [16] and possibly availability of other nutrients. Highly diverse iron sequestration mechanisms appear to play a crucial role in resistance of pink salmon to *L. salmonis*, but iron withdrawal strategy in response to lice was also reported in Atlantic salmon [16]. This study suggested that modulation of this line of defense could be achieved by nutrition. In line with the proposal that sequestration of iron away from lice constitutes an aspect of protection, we observed increased expression levels of several genes coding for iron carrying and heme-binding proteins in groups exposed to GLs, including serotransferrin 1 and 2 in I-HD, and metalloreductase STEAP4 in I-LD (Fig. 2d, Table 2). In further support of this view, findings presented in a related paper (Stanko Skugor, personal communication) outlined the role of liver, muscle and distal kidney in iron sequestration in Atlantic salmon fed GLs-containing feeds.

qPCR analysis confirmed higher expression in I-LD and I-HD groups of several pro-inflammatory cytokines, chemokines and effectors, including the neutrophil attractant *LECT2* and cytokines *IFN*γ and *IL17A* involved in Th1 and Th17-guided immune responses in mammals (Fig. 4a, b). Preconditioning naïve salmon skin by feeding GLs appears to oppose suppression and modulation of host immunity by lice, as the lowest expression level of Type 1 genes was found in I-C fish at the end of the challenge trial (Fig. 2c). We also wish to draw the attention to context-dependent fine-tuning of skin responses by GLs, exemplified by *IL17A* and *MPO* regulation (Fig. 4b, c). Downregulation was observed in the not-infected group (NI-HD) and in contrast, high expression was found in GLs supplemented groups post-infection (I-LD and I-HD). This context-dependent regulation (in absence *vs* in presence of infection) indicates that the preconditioning by GLs acts at a level other than the effector/mediator molecules MPO and IL17A (e.g. at the level of sensors or adaptors). Additional studies are needed to understand this in more detail.

While induction of anti-inflammatory mediators and ECM components (e.g. collagens) involved in strengthening of the physical skin barrier characterised I-C group, the best-protected group (I-LD) showed the opposite response: highest induction of extracellular *MMPs* involved in digestion of collagens and other ECM proteins (Table 2). Marked upregulation of *MMPs* have been observed around lice attachment sites in Atlantic salmon [11, 17, 18, 40], and importantly, even more so in the resistant pink salmon 48 h post-infection [11]. Together with *MMPs*, a number of immune genes was most highly expressed in I-LD and I-HD groups. The group included cathelicidin antimicrobial peptide 2 (Table 2), previously implied in estrogen-mediated protection [39] and found to be responsive in stock bred for increased lice resistance [17], as well as two other genes coding for antimicrobial proteins, namely granzyme A and natterin-like protein. These findings suggest that host-interactions with or the skin microbiome *per se* could play a role in resistance to lice, which may open an exciting new field for future investigations. Serum amyloid is induced early in skin and spleen during *L. salmonis* infection in Atlantic salmon [40]. The relevance of serum amyloids as candidates of protection in this study (Fig. 2d) is underscored by observations of suppressed serum amyloid A in skin of susceptible species, and activation in resistant species [11, 12, 16]. Immune mediators might direct some of the observed changes in the group of genes governing tissue turnover of nutrients, e.g. expression of fatty acid-binding protein (Table 3), which was 4-fold higher in control compared to other infected groups, is regulated by Th2 cytokines IL4 and 13 in humans [63].

Co-regulation of genes encoding myofiber proteins and multiple glycolytic enzymes is a hallmark of transcriptomic changes observed in our previous studies [18, 38, 40], suggesting that contractions in lice infected skin are fueled by glycolytic oxidation of sugars. Suppression of contractile activity in GLs-fed groups implied in this study could be associated with protection against lice. Downregulation of a number of genes encoding lipid metabolism and tissue differentiation regulators and ECM components (Table 3) in fish exposed to GLs also deserves attention, as it likely affected composition and consequently physicochemical properties of skin and mucus. Dietary GLs treatment could result in skin and mucus becoming less nutritious for lice. Such changes could also interfere with the host recognition by *L. salmonis* and the ensuing fast attachment of parasites to skin. Lipids present in fish mucus play a significant role in defining its viscosity [64] with obvious consequences for the lice attachment process. Moreover, it has been known for a long time in mammals that lipid sebum extracts contain volatile host odor components involved in the attraction of parasites [65]; thus, changes in the lipid content or lipid composition might have the potential to affect retention/formation of host kairomone semi-chemicals in Atlantic salmon mucus. To what extent this plays a role in early or late stages of sea lice infection remains unknown, and additional studies are needed to better understand the underlying mechanisms.

Finally, the present study suggested that GLs-mediated suppression of sex hormone-binding globulin beta, involved in regulation of sex hormone levels [66], may have increased the availability of steroid hormones in protected fish (Table 3). The sex steroid hormonal system in fish skin is important in wound healing in sea bream [67] and is associated with protection against lice in Atlantic salmon [39]. Future feeding studies could explore the possibility to specifically promote beneficial expression of sex steroid hormones in skin while avoiding adverse hormonal effects in non-target tissues.

## Conclusions

Feeding Atlantic salmon anti-attachment feeds containing GLs resulted in skewing of inflammatory responses towards Type 1 immunity, including gene expression programs centred on interferons in the skin prior to infection. Such dietary preconditioning seems beneficial upon encounter with the parasite as activation and maintenance of Type 1 immune genes coincided with the reduction in lice numbers. In addition, GLs-mediated gene expression changes implicated in the physicochemical properties of skin and mucus and metabolism of nutrients (iron, lipids and sugar) might interfere with the host recognition, attachment process and development of the parasite.

### Data accessibility

Microarray gene expression data files have been deposited to Gene Expression Omnibus (GSE79393).

## Additional file

Additional file 1:
**Table S1.** Primers used for real-time qPCR analysis. **Table S2.** Formulation and proximate compositions of the feeds. (DOCX 23 kb)
