# Supplementary material for: Dietary phytochemicals modulate skin gene expression profiles and result in reduced lice counts after experimental infection in Atlantic salmon
Source: Parasit Vectors. 2016 May 10;9:271. doi: 10.1186/s13071-016-1537-y (PMC4862074; doi:10.1186/s13071-016-1537-y)
Supplement: Additional file 1: — Table S1. Primers used for real-time qPCR analysis. Table S2. Formulation and proximate compositions of the feeds. (DOCX 23 kb) [file 13071_2016_1537_MOESM1_ESM.docx]

**Additional file 1**

**Table S1** Primers used for real-time qPCR analysis. Efficiencies of all primer pairs ranged from 1.80-2.00

| Gene name and symbol | GenBank | | Sequence |
| --- | --- | --- | --- |
| Barrier to autointegration factor 1 (*BANF*) | BT049316.2 | | F: ACAGACCCCTCATCATCCTG |
|  | | | R: CGGTGCTTTTGAGAAGTGGT |
| Cathelicidin-derived antimicrobial peptide 2 | AY542961.1 | | F:CCTCTTCTTGTCCGAATCTTCT |
| (Cathelicidin) |  | | R:ACACCCTCAACACTGACC |
| Complement C3 (*C3*) | L24433.1 | | F: GAGGAAAGGTGAGCCAGATG |
|  | | | R: TGTGTGTGTCGTCAGCTTCG |
| *CXCL10* | AJ417078.1 | | F: CAGGTGGGTCATTCTAAAGC |
|  | | | R: CTTGGCAAATGGAGCTTCTG |
| Elongation factor-1α (*ef1a*) | BT072490.1 | | F: GCTGTGCGTGACATGAGG |
|  | | | R: ACTTTGTGACCTTGCCGC |
| Interferon γ (*IFNγ*) | FJ263446/AY795563 /AY795563 | | F: CTAAAGAAGGACAACCGCAG |
|  | | | R: CACCGTTAGAGGGAGAAATG |
| Interleukin 4/13 (*IL4/13*) | AB574339 | | F: GACCACCACAAAATGCAAGGA |
|  | | | R: GGTTGTCTTGGCTCTTCAC |
| Interleukin 8 (*IL8*) | NM_0011407102 | | F: ATTGAGACGGAAAGCAGACG |
|  | | | R: CGCTGACATCCAGACAAATCT |
| Interleukin 17A (*IL17A*) | | GW574233 | \| F:TGGTGTGTGCTGTGTGTCTATGC \| Fwd \| TGGTGTGTGCTGTGTGTCTATGC \| \| --- \| --- \| --- \| \| GW574233 \| Rev \| TTTCCCTCTGATTCCTCTGTGGG \| |
|  | | | R:TTTCCCTCTGATTCCTCTGTGGG |
| Leukocyte cell-derived chemotaxin 2 (*LECT2*) ((LECT2) | | BT050009.2 | F: TGTGGTGCTCATAGCTGT |
|  | | | R: CTGTCCTCCTCCTGTTACT |
| Myeloperoxidase (*MPO*) | | BT072012.1 | F: TGCCATGTTCCCCAATGT |
|  | | | R: CGATACCACCCTCAAAAACT |
| Zymogen granule membrane protein 16 (*ZG16*) | [BT057545.1](http://www.ncbi.nlm.nih.gov/nucleotide/221221511?report=genbank&log$=nucltop&blast_rank=1&RID=S8HU5YS101R) | | F: GTTGAGGTGTCTGGGAAGT |
|  |  | | R:GTTGGCTGGGTAGAAGTTG |

**Table S2** Formulation and proximate compositions of the feeds

| **Ingredients (%)** | **Control** | **LD** | **HD** |
| --- | --- | --- | --- |
| Marine proteins | 49.00 | 47.22 | 42.61 |
| Vegetable proteins | 20.57 | 19.84 | 17.89 |
| Oils | 23.70 | 22.84 | 20.61 |
| Binders (starch sources) | 6.00 | 5.78 | 5.22 |
| Vitamins and minerals | 0.24 | 0.23 | 0.21 |
| Others^a^ | 0.50 | 0.48 | 0.43 |
| Masking Compounds | 0.00 | 3.61 | 13.04 |
| **Total** | **100** | **100** | **100** |
| *Proximate composition* |  | | |
| Glucosinolate (expected (umol/g) | 0,00 | 7.30 | 26.35 |
| Crude protein, % | 42.16 | 42.44 | 43.19 |
| Fat, % | 25.00 | 24.09 | 21.74 |
| Soluble protein % | 8.64 | 8.64 | 8.64 |
| Dry matter % | 93.53 | 93.53 | 93.53 |
